# Hygiene and Safety of Hard Cheese Made from Raw Cows’ Milk

**DOI:** 10.3390/vetsci9100569

**Published:** 2022-10-16

**Authors:** Ioannis Sakaridis, Evdoxios Psomas, Maria-Anastasia Karatzia, Georgios Samouris

**Affiliations:** 1 Department of Hygiene and Technology of Food of Animal Origin, Veterinary Research Institute, Hellenic Agricultural Organization-Demeter, Campus of Thermi, 57001 Thessaloniki, Greece; 2 Research Institute of Animal Science, Hellenic Agricultural Organization-Demeter, 58100 Giannitsa, Greece

**Keywords:** raw cheese, hard cheese, safety, hygiene

## Abstract

**Simple Summary:**

Raw cheeses have gained the preference of some consumers because of their intense and stronger taste compared with that of pasteurised cheeses. The pasteurization of raw milk causes the inactivation of the pathogenic and beneficial microorganisms of milk and of enzymes such as proteases and lipases which play a significant role in enhancing the unique flavour of raw cheeses. This study was conducted to evaluate the microbiological status of cheese made from unpasteurized cows’ milk, to examine the safety of the cheese and to observe the changes that occurred in its microbial community during ripening and storage. The microbiological quality of raw milk was found generally good and was improved throughout the experiments. For the cheese samples, a small increase in the prevalence of indicator microorganisms in curd and cheese samples was observed for the first few days, followed by a relatively stable condition as manufacturing proceeded and throughout the ripening of the final product. The outcome of our study was that the use of good-quality raw milk under sanitary conditions, the application of good manufacturing practices and a maturation period in a controlled environment were found to be the necessary prerequisites for the production of safe raw cheese products.

**Abstract:**

This study was conducted to evaluate the microbiological status of cheese made from unpasteurized cows’ milk, to examine the safety of the cheese and to observe the changes that occurred in its microbial community during ripening and storage. Furthermore, the pH, the moisture and salt concentration were also monitored throughout processing, ripening and storage. Seven cheesemaking trials took place along with the microbiological and physicochemical analysis of the milk, curd and cheese produced. The milk used for the cheesemaking, two curd samples before the heating and two after the heating, two cheese samples at days 3, 7, 15, 30, 60 and 90 were subjected to microbiological analysis for total mesophilic bacterial count (for milk only), Enterobacteriaceae, *E. coli, Staphylococcus*, *Salmonella*, *Listeria*, and *Clostridium*. The microbiological quality of raw milk was found to be good. It was initially slightly above the EU limit but improvements associated with farm biosecurity and milking equipment hygiene led to a significantly improved milk quality. A small increase in the prevalence of indicator microorganisms in curd and cheese samples was observed for the first few days, followed by a relatively stable condition as manufacturing proceeded and throughout the ripening of the final product. In two cheesemaking trials, *Clostridium perfringens* and *Salmonella* spp. were detected, the first originating from the milk and the second from the environment. The use of good-quality raw milk under sanitary conditions, the application of good manufacturing practices and a maturation period in a controlled environment were found to be the necessary prerequisites for the production of safe raw cheese products.

## 1. Introduction

Cheese is one of the oldest known types of processed foods and was initially used as a way to concentrate and preserve milk [1]. In the past, the cheese making process and the ripening of cheese could partially control the growth of pathogenic bacteria and cheese was a relatively safe food to store and consume, despite the absence of refrigeration equipment. Later on, the discovery of pasteurisation led cheese producers to subject milk to a thermal processing procedure equivalent to pasteurisation in order to eliminate the pathogenic bacteria present in raw milk and to make cheese safer for consumers. However, there are a number of cheeses that were and still are traditionally made from unpasteurised milk and are very sought after by the consumers. Raw cheeses have gained the preference of some consumers because of their intense and stronger taste compared with that of pasteurised cheeses [2]. This unique taste is a consequence of complex metabolic activities that take place during the processing and maturation of the cheese [3]. Raw cheeses comprise a significant number of volatile compounds, such as carboxylic acids, esters and alcohols, coming from the fermentation of milk components by the natural microbiota of raw milk [4]. The microbiota of raw cheeses consists of a large number of beneficial bacteria, such as *Lactococcus* spp., *Lactobacillus* spp., *Leuconostoc* spp. and *Enterococcus* spp. [2,5]. The pasteurisation of raw milk causes the inactivation of these microorganisms and of enzymes such as proteases and lipases which play a significant role in enhancing the unique flavour of raw cheeses [6]. The texture of raw cheeses can also be differentiated according to the composition of the microbial community of raw milk, along with the processing and seasonal conditions of cheese making [7,8]. Therefore, the multiplicity of the microbiota of raw milk can significantly enhance the flavour and texture of raw cheeses and produce cheeses with distinct and desirable characteristics, compared to the pasteurised cheeses.

The microbiological safety of raw cheeses is a highly controversial topic. There are several studies that report a large number of food-borne outbreaks that were related to the consumption of unpasteurised cheeses. In the United States, from 2009 until 2014, unpasteurised dairy products were found to be responsible for almost all of the 761 illnesses and 22 hospitalisations that occurred annually because of dairy-related outbreaks caused by STEC, *Salmonella* spp., *L. monocytogenes* and *Campylobacter* spp. [9]. The large majority of these illnesses, more than 95%, were salmonellosis and campylobacteriosis. The same study [9] reports that although the consumers of unpasteurised milk and cheese are a small fraction of the US population, they are 838.8 times more likely to experience an illness and 45.1 times more likely to be hospitalised, compared with consumers of pasteurised products. However, other studies report that pasteurised cheeses caused outbreaks of food-borne illness, and in some cases at a higher incidence rate than unpasteurised cheeses [10]. Other researchers stated that an extremely low or zero percentage of raw milk cheeses have been contaminated with major pathogens including *Listeria monocytogenes* [11]. This comes in accordance with the findings of a later study in the US, where illnesses caused by *L. monocytogenes* were found to be associated with the consumption of pasteurised cheese more often, albeit only causing one additional outbreak of a related illness per year on average [9]. The antagonistic activity of the microbiota of raw cheese probably obstructs the growth of some pathogens such as *L. monocytogenes*, but the bacteria responsible for growth inhibition and their mechanism have not been found yet [5]. Similarly, other researchers reported that foodborne pathogens such as *L. monocytogenes*, *Salmonella* spp. and *S. aureus* were barely isolated from raw milk and soft cheese, thanks to the antagonistic activity of indigenous lactic acid bacteria [12].

The aim of the present study was to evaluate the microbiological status of cheese made from unpasteurised cows’ milk, to examine the safety of the cheese and to observe the changes of its microbial community during ripening and storage. In addition to that, the pH, the moisture and salt concentration were also monitored throughout processing, ripening and storage.

## 2. Materials and Methods

### 2.1. Cheese Making Process

Raw cows’ milk was transferred from cooling tanks to an open vat (batch pasteurizer) after it was filtered for the presence of foreign particles. The milk was heated to 35–36 °C to add the rennet (2000 Powder Rennet, Paride Venturelli S.r.l., Bagnolo Cremasco, Italy). No defined starter cultures were added to this batch (batch-R). At the same time, another batch of raw milk (batch-P) was pasteurised (63 °C for 30 min) and then cooled to 40 °C for the addition of the starter culture (*Lactococcus lactis* subsp. *lactis* biovar *diacetylactis*, *Leuconostoc mesenteroeides* subsp. *mesenteroeides*, *Streptococcus thermophilus* sbsp. *salivarius*, *Lactobacillus delbrueckii* subsp. *bulgaricus*, *Lactobacillus helveticus*) (MALP721, Mediterranea Biotechnologie S.r.l., Termoli, Italy). After 30 min the milk was cooled to 35–36 °C for the addition of CaCl_2_ (0.2%) and rennet (2000 Powder Rennet, Paride Venturelli S.r.l.). After about 35–40 min from the addition of rennet, milk of both batches (R and P) was coagulated. The formed curd was then cut into small pieces (1 cm × 1 cm × 1 cm size) and was cooked under stirring for 20–30 min at a temperature of 50–52 °C in order to expel the whey. The curd was then inserted into moulds, followed by pressing (1–2 times their weight) and was inverted for uniform moulding-drainage to shape the cheese, for 1–2 h. After 24 h and when the pH of the cheese reached 5.4, the cheeses were immersed into brine 18–20 Be at a temperature of 13–14 °C. For each kilo of cheese, 8–9 h of immersion in the above brine were needed. The cheeses were left to mature at 13–14 °C for 10–15 days with regular inversions every 1–2 days. After the end of the first maturation the cheeses were closed in vacuum and kept in the refrigerator (6–8 °C) for at least 3 months (2nd maturation). The cheese was ready for consumption after 3 months. The cheese making trials along with the microbiological and physicochemical analysis of the curd and cheese were repeated 7 times in total.

### 2.2. Milk Samples

Milk samples were collected from the cooling tanks of a commercial Holstein dairy farm. Four milk samples were obtained during each sampling and the sampling was repeated seven times. The rolling geometric average was calculated for all milk samples.

### 2.3. Curd and Cheese Samples

A total of four curd samples were obtained during the day of the cheese making process, two before and two after the cooking and during the moulding for each batch (raw and pasteurised cheese). Two samples of cheese from each batch were collected 2 days after salting, leading to an initial microbial selection. Two cheese samples at different ripening stages were also collected for each batch at 7 days, 15 days, 30 days, 60 days and 90 days of ripening. 

During the 90 days ripening of cheeses, qualitative formation of gas and off-odours were regularly monitored, by visual inspection and by smell. Cheeses with EBD (early blowing defect) that showed blown-packaging, irregular eyes, cracks or splits, as a consequence of the cheese matrix notwithstanding the pressure of produced gas, and unpleasant or rancid odour were removed and further investigated for the presence of gas forming bacteria. 

All samples were transported to the laboratory under refrigerated conditions not later than 1h after the collection and were subjected to microbiological analysis.

### 2.4. Microbiological Analysis

Decimal dilutions of raw and pasteurised milk were prepared in peptone water (0.1% mycological peptone, Oxoid, Basingstoke, UK). Dilutions were plated and incubated as follows: total mesophilic microflora on PCA incubated aerobically at 30 °C for 24 h (ISO 4833-1:2013); *Salmonella* on XLD agar incubated at 37 °C for 24 h (ISO 6579:2002); *Listeria* spp. and *monocytogenes* on ALOA incubated at 37 °C for 48 h (ISO 11129-2:2017); Enterobacteriaceae on violet red bile glucose agar (VRBG agar) incubated at 37 °C for 24 h (ISO 21158-2:2017); *Escherichia coli* on Tryptone bile x-glucuronide agar (TBX agar) incubated at 44 °C for 24 h (ISO 16649-2:2001); *Staphylococcus* spp. on Baird–Parker agar incubated at 37 °C for both 24 h and 48 h (ISO 6888-1/2:1999), *Clostridium perfringens* on Tryptose Sulfite Cycloserine Agar incubated at 37 ± 1 °C for 18–24 h under anaerobic conditions. All analyses were performed for the identification and enumeration of the pathogens. All media were purchased from Oxoid (UK).

For microbiological analysis, 25 g of curd and cheese samples were emulsified with sterile quarter strength Ringer solution (Oxoid, UK) in a stomacher machine. Decimal dilutions were prepared in Ringer solution and plated onto different selective media for viable counts. The following analyses were carried out: *Salmonella*; *Listeria* spp. and *monocytogenes*; Enterobacteriaceae; *Escherichia coli*; *Staphylococcus* spp. and *Clostridium perfringens*, all as described above.

During the second cheesemaking trial and after the salting step, small eyes with smooth and bright inners were detected and the samples were screened for the presence of spore forming bacteria. The cheese samples (cheeses after salting, 25 g) were aseptically transferred into a stomacher bag and 225 mL of 0.1% buffered peptone water (BPW; Oxoid, UK) were added and homogenised for 120 s (BagMixer, USA). One millilitre of homogenate was then plated on Tryptose Sulfite Cycloserine Agar (TSC, Liofilchem, Italia), followed by overlaying 10 mL egg yolk free TSC over the TSC. The colonies were counted manually after incubation at 35 °C for 24 h [13].

All media were purchased from Oxoid.

### 2.5. pH, Moisture and NaCl Content

The pH of cheese was measured with an electronic Consort pH-meter (Turnhout, Belgium). The moisture content of cheese samples (2–5 g) was determined after drying at 105 °C until constant weight (ISO—ISO 2920:2004). Results were expressed as percentage (%). The modified Volhard test [14] was used to determine the NaCl content. Duplicate analysis was performed for all parameters in consideration.

### 2.6. Statistical Analysis

Statistical analysis was carried out using Statistical Software (IBM SPSS Statistics 23, SPSS Inc., Chicago, IL, USA). The Kruskal–Wallis H test was used to compare cheese samples manufactured using raw and pasteurised milk. *p* ≤ 0.05 was considered statistically significant. Kruskal–Wallis H test was performed for Enterobacteriaceae, *E. coli* and *Staphylococcus* levels (CFU/g) in cheese samples manufactured using raw and pasteurised milk, for each manufacturing stage (before reheating, after reheating and on days 3, 7, 15, 30, 60 and 90). The mean and standard error of the parameters under study were calculated both for the milk used for cheese production, as well as for curd and cheese samples.

## 3. Results

### 3.1. Milk Samples

The total mesophilic bacterial counts in raw milk ranged between 3.62 and 5.67 log10 cfu/mL. A relatively high prevalence of Enterobacteriaceae was observed in some of the raw milk samples with the geometric means ranging between 2.2 and 5.69 log10 cfu/mL. A significant prevalence of *Staphylococcus* spp. was observed in most of the raw milk samples with the geometric means ranging between 2.362 and 4.38 log10 cfu/mL. The detailed results from the microbiological analysis of the raw milk samples are shown in the following Table 1.

### 3.2. Curd and Cheese Samples

**Figure 1:**
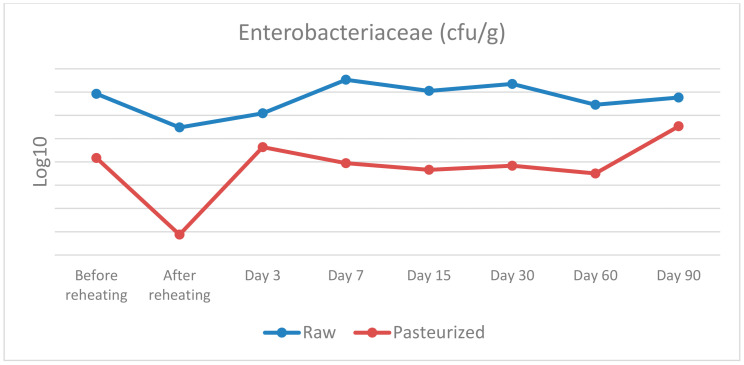
Enterobacteriaceae counts in raw and pasteurized curd and cheese samples.

**Figure 2:**
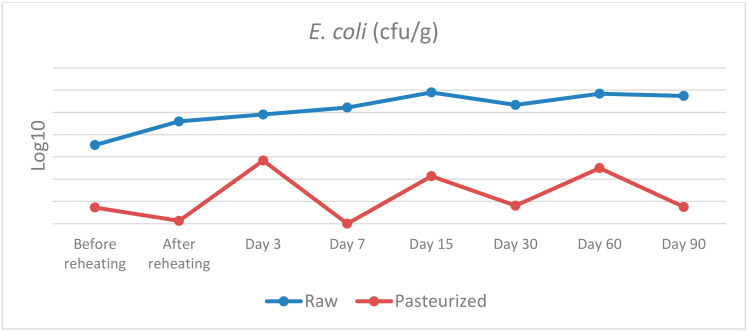
*E. coli* counts in raw and pasteurized curd and cheese samples.

**Figure 3:**
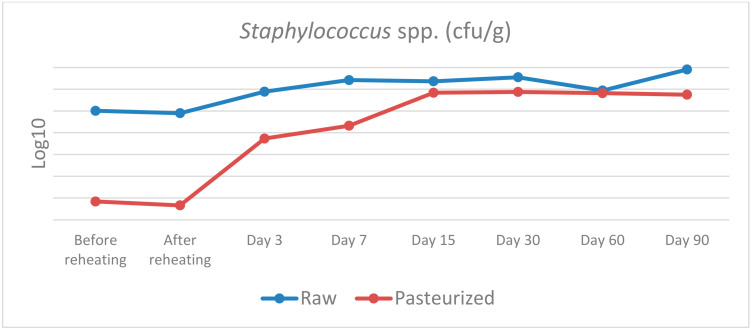
*Staphylococcus* spp. counts in raw and pasteurized curd and cheese samples.

The results of the microbiological analysis of raw and pasteurized curd and cheese samples during the cheesemaking trials and the maturation are presented in Table 2 and Figure 1, Figure 2 and Figure 3.

The statistical interpretation of the microbiological analysis of the raw and pasteurised curd and cheese samples during the cheesemaking trials and the maturation is shown in Table 3. 

Enterobacteriaceae: before cooking, a statistically significant difference was found between raw or pasteurised curd, χ^2^(1) = 5.333, *p* = 0.021, with a mean rank Enterobacteriaceae level of 6.5 for raw and 2.5 for pasteurised curd. After cooking, a similar statistically significant difference was observed, χ^2^(1) = 5.398, *p* = 0.020, with a mean rank Enterobacteriaceae level of 6.5 for raw curd and 2.5 for pasteurised curd. On day 7, the statistically significant difference detected was χ^2^(1) = 3.857, *p* = 0.050, with a mean rank Enterobacteriaceae level of 5.0 for raw cheese and 2.0 for pasteurised cheese. On days 15 and 30, a similar trend of statistically significant difference was discovered, with χ^2^(1) = 5.333, *p* = 0.021, with a mean rank Enterobacteriaceae level of 6.5 for raw cheese and 2.5 for pasteurised cheese, in both sampling days. Finally, on day 60, a statistically significant difference was noted, with χ^2^(1) = 4.133, *p* = 0.042, with a mean rank Enterobacteriaceae level of 6.25 for raw cheese and 2.75 for pasteurised cheese. No significant differences in Enterobacteriaceae levels were found between raw and pasteurised groups on days 3 and 90. 

*E. coli*: before cooking, a statistically significant difference was found between raw and pasteurised curd, χ^2^(1) = 5.398, *p* = 0.020, with a mean rank *E. coli* level of 6.5 for raw and 2.5 for pasteurised curd. On day 7, the statistically significant difference detected was χ^2^(1) = 4.355, *p* = 0.037, with a mean rank *E. coli* level of 6.0 for raw cheese and 3.0 for pasteurised cheese. On days 30 and 60, a similar trend of statistically significant difference was discovered, with χ^2^(1) = 4.133, *p* = 0.042, with a mean rank of 6.25 for raw cheese and 2.75 for pasteurised cheese, in both sampling days. Finally, on day 90, a statistically significant difference was noted, with χ^2^(1) = 5.600, *p* = 0.018, with a mean rank *E. coli* level of 6.5 for raw cheese and 2.5 for pasteurised cheese. No significant differences in *E. coli* levels were observed between raw and pasteurised groups after reheating and on days 3 and 15.

*Staphylococcus* spp.: before and after cooking, a statistically significant difference was found between raw and pasteurised curd, χ^2^(1) = 5.398, *p* = 0.020, with a mean rank *Staphylococcus* level of 6.5 for raw and 2.5 for pasteurised curd. No other statistically significant differences were observed at any of the remaining samplings (days 3, 15, 30, 60 and 90). Despite the relatively high levels of *Staphylococcus* spp. that were isolated in the cheese samples during maturation, *Staphylococcus aureus* was not detected. 

In the second cheesemaking trial and after the salting step, small eyes with smooth and bright inners were detected and the cheese samples were screened for the presence of spore-forming bacteria. *Clostridium perfringens* was isolated in both raw and pasteurised cheese samples. The milk that was used for this cheesemaking trial was also examined and *Clostridium perfringens* was detected in the milk samples. Therefore, the origin of *Clostridium perfringens* can be attributed to the milk used for the cheesemaking trial and not to a contamination that occurred during the cheese making process.

Moreover, *Salmonella* spp. was detected in the pasteurised milk and the raw curd before cooking during the fourth cheesemaking trial. However, in the next steps of the same trial *Salmonella* spp. was not detected. All other milk, curd and cheese samples tested were found to be free of *Salmonella* spp. Similarly, *Listeria* spp., was never detected in all samples tested.

### 3.3. pH, Moisture and NaCl Content

The pH, moisture and NaCl content of the cheese samples throughout the ripening period are shown in the following Table 4.

## 4. Discussion

### 4.1. Milk Samples

The microbiological quality of raw milk that is used for the production of raw cheese is very important and can have a critical impact on the safety of the final product. Total mesophilic bacterial counts in raw milk ranged between 3.62 and 5.67 log10 cfu/mL. The raw milk samples of the first, third and fourth experimental trials were found slightly above the legislative limit set by the EU (EC 853/2004, EC 2073/2005) of 10^5^ cfu/mL for total mesophilic bacteria of raw milk intended for cheese making. However, improvements on the farm in terms of milking process and milk storage along with the updated biosecurity measures have improved the microbiological quality of milk and this was reflected on the next three experiments where the geometric means of the raw milk samples were significantly lower and within the EU legislative limits set for raw milk intended to be used for the production of raw cheese. In more detail, in the milking parlour, teat cups that were worn were replaced, vacuum and pulsation were recalibrated to ensure correct function and post-milking teat disinfection was practiced. The temperature in milk tanks was closely monitored and cooling settings were modified to avoid temperature spikes and an increase in mean temperature. Our results are comparable with the findings of some others researchers [15] where total mesophilic bacteria were higher than 100,000 cfu/mL in 23.6% of all tested samples and its mean value in all milk samples was 4.5 log10 cfu/mL. Slightly lower counts are reported in other studies from Belgium (3.96 log10 cfu/mL [16], the United States (4.06 log10 cfu/mL [17]), and Finland (4.11 log10 cfu/mL [18]), and higher counts from China (5.10 log10 cfu/mL [19]) and Morocco (5.87 log10 cfu/mL [20]).

A relatively high prevalence of Enterobacteriaceae was observed in some of the raw milk samples with the geometric means ranging between 2.2 and 5.69 log10 cfu/mL. However, these results were lower than the ones reported in Egypt where Enterobacteriaceae were detected in 84% of examined raw milk samples (with mean count of 1.02 × 10^6^ + 1.98 × 10^5^ cfu/mL [21]) and the Czech Republic where Enterobacteriaceae counts ranged between 1.0 × 10^1^ and 2.0 × 10^6^ CFU/mL [22]. *E. coli* was detected in the raw milk samples of only two of the cheesemaking trials with low numbers (2.45 and 2.52 log cfu/mL). In the same study from the Czech Republic, the presence of *E. coli* was confirmed in 86.3% of samples and the colony counts ranged from 1.0 × 10^1^ to 4.0 × 10^6^ CFU/mL, while in Egypt the incidence of pathogenic *E. coli* in raw milk was 55% and the mean count was 3.0 × 10^4^ ± 1.3 × 10^4^ cfu/mL [23]. Enterobacteriaceae and *E. coli* presence in milk may indicate a faecal contamination of milk or can be linked to mammary infections [24].

A significant prevalence of *Staphylococcus* spp. was observed in most of the raw milk samples with the geometric means ranging between 2.362 and 4.38 log10 cfu/mL. However, *Staphylococcus aureus* was never detected in all our samples tested. *Staphylococcus aureus* is usually responsible for both clinical and subclinical mastitis in cows [25]. These infections often result in significant economic losses due to reduced milk production and represent potential causes of foodborne intoxication [22]. The occurrence of *S. aureus* in raw milk ranges from 12.4% to 75% [26,27]. In a more recent study, the presence of *S. aureus* was confirmed in 26.9% of samples but the counts were either negative or less than 5.0 × 10^2^ CFU/mL [22].

### 4.2. Curd and Cheese Samples

A total of 16 curd and cheese samples were taken for microbiological analysis during each cheesemaking trial along with a maturation period of three months. As stated in the results section, *Clostridium perfringens* was isolated from both raw and pasteurised cheese samples in the second cheesemaking trial. The cheese samples presented small eyes with smooth and bright inners along with a blowing defect. Cheeses are traditionally affected by a common paste defect known as early blowing which involves texture alterations produced by unusual gas fermentation. Such a defect is of great economic concern in cheese production as they are responsible for consumer rejection [28]. After the appearance of this defect, the milk used for this cheesemaking trial was subjected to further microbiological analysis and *Clostridium perfringens* was detected in the milk samples. Therefore, we can conclude that the milk used for the cheesemaking trial was probably at fault for the presence of *Clostridium perfringens* in the cheese samples.

Furthermore, *Salmonella* spp. was detected in the pasteurised milk and the raw curd before reheating during the fourth cheesemaking trial. However, *Salmonella* spp. was not detected in the milk that was used for this cheesemaking trial or in the following steps of the same trial. This means that it was probably a minor contamination that occurred during the cheesemaking process from either the equipment or the personnel. It is very important to highlight that *Salmonella* spp. was not detected in the following steps, which indicates that the maturation step along with the indigenous microflora or the one induced by the starter culture were able to control the presence of this pathogen.

As far as Enterobacteriaceae and *E. coli* are concerned there are several statistically significant differences between raw and pasteurised curd and cheese samples. To be more precise, statistically significant differences were found for the samples before and after reheating on days 3, 7, 15, 30 and 60 for Enterobacteriaceae, and before reheating on day 7, 30, 60 and 90 for *E. coli*. Therefore, the use of raw or pasteurised milk for cheese production seems to have an important impact on the presence of these bacteria. The maturation process does not seem to affect the prevalence of these bacteria and their population reaches a level of 7 log10 CFU/g and 6 log10 CFU/g for Enterobacteriaceae and *E. coli*, respectively, on the 90th day.

Our results are not in agreement with the findings of several studies that took place in the past concerning the microbiological status and the presence of indicator bacteria such as Enterobacteriaceae and *E. coli* in raw cheeses. During the first stages of maturation there is regularly an increase in the prevalence of indicator microorganisms, followed by a decline as the cheesemaking proceeds and throughout the maturation period of the cheese [29]. Some researchers [30] reported that a 2–3 log10 CFU/g level of Enterobacteriaceae in the raw milk increased to a level of 5–8 log10 CFU/g in the cheese after a week of maturation, whereas after 6 weeks, a 1 to 5 log10 reduction for the interior and a 2 to 4 log10 reduction on the surface was found. In another study [31] the Enterobacteriaceae counts decreased by 1.87–2.84 log10 CFU/g in five of the six samples over the 120-day ripening period and *E. coli* could not be detected after 60 days of ripening.

Moreover, there are several studies that examined the levels of indicator bacteria in raw cheeses after the ripening period. Researchers from the U.S. [32] examined 41 raw-milk cheeses made from bovine, caprine or ovine milk from the U.S. and found 95% of samples (39/41) to have < 10 CFU/g of *E. coli,* while the other two samples, a bovine and an ovine milk cheese, contained *E. coli* at 10 and 30 CFU/g, respectively. Similarly, others [33] (2009) analysed 351 cheeses, which were made from both raw or pasteurized bovine, caprine or ovine milk and reported that 79% of the raw-milk cheeses had *E. coli* at < 10 CFU/g. In another study [34], 24 samples of a Spanish cheese made with raw bovine milk were tested for *E. coli* and a mean level of 1.72 log10 CFU/g was found. In a similar study such as ours, where both raw and pasteurised milk cheeses were analysed, researchers [35] examined 151 samples (96 pasteurized-milk and 55 raw-milk cheeses) and found only 3% (3/96) of the pasteurized-milk cheeses contained *E. coli,* whereas 34% (19/55) of the raw-milk cheeses were positive for *E. coli*. However, some of these cheeses contained *E. coli* at levels of 3–5 log10 CFU/g and some of the raw milk cheeses contained *E. coli* at levels above 5 log10 CFU/g. The importance of using quality raw milk and hygienic barriers to improve process control was highlighted in this study in order to improve the safety of raw-milk cheeses.

Regarding *Staphylococcus* spp., our results indicate that there was not any statistically significant difference between raw and pasteurised cheese after the first day of the experiments, while a significant contamination was observed in both cases, reaching almost 10^8^ and 10^7^ log10 CFU/g, respectively. Fortunately, *Staphylococcus aureus* was not detected during all cheesemaking trials despite the large numbers of *Staphylococcus* spp. In our experiments, *Staphylococcus* was found to be present in raw milk and in the first few steps of the cheesemaking process of the pasteurised milk which means that was probably originated from the milking parlour and the cheesemaking equipment along with the people involved in cheese manufacturing. An increased contamination of traditionally made cows’ milk cheese with *S. aureus* has been reported by other authors, especially at the stage from milk to curd [36]. This contamination could be attributed to the physical entrapment of *S. aureus* in the curd and his ability to grow rapidly in milk [37]. Another potential source of contamination are the personnel involved in cheese manufacturing, since *S. aureus* is frequently found on the skin of cheese makers, and this could be the main source of contamination in the later stages of cheese manufacture [38].

Finally, the results from the pH, moisture and NaCl content measurements for both raw and pasteurised cheese are in accordance with the findings of other researchers in a previous study that examined similar cheese products [39]. Similar cheeses had a pH that varied between 5.42–5.50, moisture at 35.2–37.2% and NaCl content at 1.46–1.85%. The mean values of our raw and pasteurised cheese samples at day 90 were pH at 5.35/5.49, moisture at 36.36%/34.84% and NaCl content at 1.80%/1.86%, respectively. These values are typical for the cheese type produced and that is why we cannot conclude their effect on the higher than usual microbial growth observed in our cheese samples during maturation.

## 5. Conclusions

This study aimed to evaluate the microbiological status of cheese made from unpasteurized cows’ milk, to examine the safety of the cheese and to report the changes of its microbial community during ripening and storage. The raw milk samples of the first few experimental trials were found to be slightly above the legislative limits of the EU for total mesophilic bacteria of raw milk intended for cheese making, while the improvements that were applied on the farm in terms of the milking process and milk storage, combined with the updated biosecurity measures have significantly improved the microbiological quality of milk in the next trials. Besides the two cheesemaking trials where *Clostridium perfringens* and *Salmonella* were detected and the indicator microorganisms Enterobacteriaceae, *E coli* and *Staphylococcus* that were found in significant numbers in all cheese samples, we did not detect any other pathogenic bacteria that could potentially harm the health of consumers. The physicochemical properties of all cheese samples were good and consistent with similar cheeses produced in Greece from pasteurized milk.

However, taking into account the increased levels of the indicator microorganisms in both raw and pasteurized cheese samples during the cheese making process and especially the ripening period, it can be concluded that they could be attributed to contamination from the processing facility, the equipment and the personnel. Although the raw cheeses produced in our experiments were within the microbiological limits set by EU, the high level of the indicator microorganisms and the fact that their levels did not decline during ripening, generates some doubts about their safety and requires further investigation. To produce a safe raw cheese product, good quality milk that comes from a farm with high biosecurity standards, milking and processing of the milk under sanitary conditions, maturation of the cheese in controlled and hygienic environment and application of good manufacturing practices from the personnel, are necessary.

## Figures and Tables

**Table 1:** Total mesophilic bacterial count, Enterobacteriaceae, *E. coli*, and *Staphylococcus* spp. counts in raw milk samples (geometric mean).

	Total Mesophilic Bacterial Count (cfu/mL)		Enterobacteriaceae (cfu/mL)		*E. coli* (cfu/mL)		*Staplylococcus* spp. (cfu/mL)	
Sampling	Geometric Mean (cfu/mL)	Log10 (cfu/mL)	Geometric Mean (cfu/mL)	Log10 (cfu/mL)	Geometric Mean (cfu/mL)	Log10 (cfu/mL)	Geometric Mean (cfu/mL)	Log10 (cfu/mL)
1	3.9 × 10^5^	5.59	3.5 × 10^5^	5.54	N/D	N/D	2.4 × 10^4^	4.38
2	4.2 × 10^3^	3.62	6.7 × 10^2^	2.83	N/D	N/D	5.0 × 10^2^	2.70
3	4.1 × 10^5^	5.61	4.9 × 10^5^	5.69	N/D	N/D	N/D	N/D
4	4.7 × 10^5^	5.67	2.1 × 10^4^	4.32	2.8 × 10^2^	2.45	1.0 × 10^4^	4
5	5.0 × 10^3^	3.70	3.6 × 10^3^	3.56	3.3 × 10^2^	2.52	1.0 × 10^4^	4
6	9.6 × 10^3^	3.98	N/D	N/D	N/D	N/D	2.3 × 10^2^	2.36
7	2.9 × 10^4^	4.46	1.6 × 10^2^	2.20	N/D	N/D	5.4 × 10^3^	3.73

N/D: not detected, cfu/mL: colony forming units per ml.

**Table 2:** Enterobacteriaceae, *E. coli*, and *Staphylococcus* spp. counts in raw and pasteurized curd and cheese samples (geometric mean).

	***Enterobacteriaceae* (CFU/g**)							
	Before cooking	After cooking	Day 3	Day 7	Day 15	Day 30	Day 60	Day 90
	Mean	Mean	Mean	Mean	Mean	Mean	Mean	Mean
Raw	8.40 × 10^6^	3.04 × 10^5^	1.22 × 10^6^	3.38 × 10^7^	1.12 × 10^7^	2.23 × 10^6^	2.85 × 10^6^	5.30 × 10^9^
Pasteurized	1.47 × 10^4^	7.56 × 10^6^	4.30 × 10^4^	8.81 × 10^3^	1.07 × 10^3^	6.84 × 10^3^	3.18 × 10^3^	3.75 × 10^5^
	***E. coli* (CFU/g)**							
	Before cooking	After cooking	Day 3	Day 7	Day 15	Day 30	Day 60	Day 90
	Mean	Mean	Mean	Mean	Mean	Mean	Mean	Mean
Raw	4.00 × 10^4^	2.88 × 10^3^	8.07 × 10^4^	1.66 × 10^5^	7.88 × 10^5^	2.17 × 10^5^	6.91 × 10^5^	5.48 × 10^5^
Pasteurized	5.29 × 10^0^	7.50 × 10^−1^	6.84 × 10^2^	ND	1.62 × 10^3^	6.43 × 10^0^	3.13 × 10^2^	7.50 × 10^−1^
	***Staphylococcus* (CFU/g)**							
	Before cooking	After cooking	Day 3	Day 7	Day 15	Day 30	Day 60	Day 90
	Mean	Mean	Mean	Mean	Mean	Mean	Mean	Mean
Raw	7.38 × 10^5^	1.06 × 10^6^	7.69 × 10^6^	2.62 × 10^7^	2.29 × 10^7^	3.53 × 10^7^	8.63 × 10^6^	8.01 × 10^7^
Pasteurized	6.89 × 10^1^	4.62 × 10^1^	5.37 × 10^4^	2.08 × 10^5^	6.87 × 10^6^	7.48 × 10^6^	6.56 × 10^6^	7.08 × 10^6^

**Table 3:** Statistical interpretation of the microbiological analysis of raw and pasteurized curd and cheese.

	***Enterobacteriaceae* (CFU/g)**																													
	Before cooking					After cooking					Day 7					Day 15					Day 30					Day 60				
χ^2^	5.333					5.398					3.857					5.333					5.333					4.133				
df	1					1					1					1					1					1				
Asymp. Sig.	0.021					0.020					0.050					0.021					0.021					0.042				
Mean rank raw	6.5					6.5					5.0					6.5					6.5					6.25				
Mean rank past.	2.5					2.5					2.0					2.5					2.5					2.75				
	***E. coli* (CFU/g)**																													
	Before cooking						Day 7						Day 30						Day 60						Day 90					
χ^2^	5.398						4.355						4.133						4.133						5.600					
df	1						1						1						1						1					
Asymp. Sig.	0.020						0.037						0.042						0.042						0.018					
Mean rank raw	6.5						6.0						6.25						6.25						6.5					
Mean rank past.	2.5						3.0						2.75						2.75						2.5					
	***Staphylococcus* (CFU/g)**																													
	Before cooking															After cooking														
χ^2^	5.398															5.398														
df	1															1														
Asymp. Sig.	0.020															0.020														
Mean rank raw	6.5															6.5														
Mean rank past.	2.5															2.5														

**Table 4:** Mean values of pH, moisture and NaCl content of the cheese samples during the ripening period of the seven cheesemaking trials.

Physicochemical Parameters	Cheese	Ripening Period (days)					
		1	7	15	30	60	90
pH	Raw	5.41	5.29	5.33	5.25	5.38	5.35
	Pasteurised	5.51	5.25	5.24	5.46	5.43	5.49
Moisture (%)	Raw	41.73	38.89	38.45	38.72	36.82	36.36
	Pasteurised	45.52	40.61	38.00	34.39	35.30	34.84
NaCl (%)	Raw	1.22	1.09	1.08	1.66	2.01	1.80
	Pasteurised	1.01	1.34	1.28	1.64	1.80	1.86

## Data Availability

Not applicable.
